# An evaluation of the impact of the implementation of the Tele-ICU: a retrospective observational study

**DOI:** 10.1186/s40560-023-00657-4

**Published:** 2023-03-07

**Authors:** Taro Watanabe, Koichi Ohsugi, Yuri Suminaga, Masayuki Somei, Kazuki Kikuyama, Maiko Mori, Hiroko Maruo, Nao Kono, Toru Kotani

**Affiliations:** 1 Department of Intensive Care Medicine, Showa University School of Medicine, 1-5-8, Hatanodai, Shinagawa-ku, Tokyo, 142-8666 Japan; 2 Department of Nursing, Showa University Hospital, Tokyo, Japan

**Keywords:** Telemedicine, Intensive care unit, Telemedicine intensive care unit, Quality of health care, Workload

## Abstract

**Background:**

The telemedicine intensive care unit (Tele-ICU) is defined as a system in which intensive care professionals remotely provide care to critically ill patients and support the on-site staff in the intensive care unit (ICU) using secured audio–video and electronic links. Although the Tele-ICU is expected to resolve the shortage of intensivists and reduce the regional disparities in intensive care resources, the efficacy has not yet been evaluated in Japan because of a lack of clinically available system.

**Methods:**

This was a single-center, historical comparison study in which the impact of the Tele-ICU on ICU performance and changes in workload of the on-site staff were evaluated. The Tele-ICU system developed in the United States was used. Data for 893 adult ICU patients before the Tele-ICU implementation and for all adult patients registered in the Tele-ICU system from April 2018 to March 2020 were abstracted and included. We investigated ICU and hospital mortality and length of stay and ventilation duration after the Tele-ICU implementation in each ICU, and compared between pre and post implementation and changes over time. We also assessed physician workload as defined by the frequency and duration of access to the electronic medical record (EMR) of the targeted ICU patients.

**Results:**

After the Tele-ICU implementation 5438 patients were included. In unadjusted data pre/post study showed significant decreases in ICU (8.5–3.8%) and hospital (12.4–7.7%) mortality and ICU length of stay (*p* < 0.001), and those values were maintained for 2 years. In data stratified by predicted hospital mortality, ICU and hospital actual mortality in high and medium risk patients decreased significantly after the implementation. Ventilation duration was shortened (*p* < 0.007). Access frequency of the on-site physicians decreased by 25%, and the decrease occurred in the daytime shift and in the physicians with 3–15 years of work experience.

**Conclusions:**

Our study showed the Tele-ICU implementation was associated with lower mortality, especially in medium and high risk patients, and decreased EMR-related tasks of on-site physicians. These results suggest that the Tele-ICU could be a solution of the shortage of intensivists and regional disparities for intensive care.

**Supplementary Information:**

The online version contains supplementary material available at 10.1186/s40560-023-00657-4.

## Background

The telemedicine intensive care unit (Tele-ICU) is defined as a system in which intensive care professionals remotely provide care to critically ill patients and support the on-site staff in the intensive care unit (ICU) using secured audio–video and electronic links. Although the effectiveness of Tele-ICU varied by facility [1], a systematic review showed that intensivist staffing was inversely correlated with in-hospital mortality [2], and Tele-ICU decreased the risk of ICU and hospital mortality when compared with conventional critical care [3]. To date, it has been used for approximately 18% of all ICU patients in the United States and has been reported to have medical and economic benefits, such as reduced mortality and length of stay (LOS) in the ICU [4].

In Japan, the increase in elderly critically ill patients in addition to the declining birth rate will increase ICU utilization in the future. In total, 6911 beds are prepared for intensive care, showing that Japan has 5.6 beds for 100,000 people, and the number of beds varies from 1.5 to 11.8 among prefectures. The total number of board-certified intensivists was 2115 as of August 4, 2021, and they were concentrated in large cities [5]. The shortage of intensivists and prevalence of regional disparities in intensive care resources is reflected in the fact that only 26% of ICUs in Japan are ‘closed’ ICUs in which intensivists determine the treatment, and 50% are ‘mandatory’ ICUs in which intensivists are involved in all the treatments. The remaining 24% are ‘elective’ ICUs in which intensivists respond at the request of the attending physician [6]. Given the facts mentioned above, the Tele-ICU is expected to resolve the shortage of intensivists and reduce the regional disparities in intensive care resources. However, a system that could become clinically available has not been developed.

In 2018, intensivists at Showa University introduced the Tele-ICU system that was developed in the United States in the clinical setting. Because there are many differences between the United States and Japan, such as language, authorization of each occupation, and the healthcare and insurance system, it is necessary to verify whether the Tele-ICU system that was developed in the United States could fit the Japanese healthcare system and achieve similar outcomes. In addition, it should be assessed whether the Tele-ICU system can influence the workload of on-site ICU physicians.

This was a single-center, historical comparison study in which the impact of the Tele-ICU on ICU performance and changes in workload of the on-site staff were evaluated. We compared the patient background, ICU and hospital mortality and LOS before and after the implementation of the Tele-ICU to evaluate clinical efficiency. Staff workload was defined by the on-site physician’s frequency and total duration of access to the electronic medical record (EMR), because it has been reported that the use of EMR is directly associated with physician fatigue and burnout [7, 8].

## Methods

### Setting

Showa University Hospital is a tertiary medical education facility in Tokyo, and the Tele-ICU supports 31 beds in three ICUs (surgical/medical, cardiac, emergency) that belong to the university hospital and 15 beds in a surgical/medical ICU of the regional hospital located 15 km away. The support center, Showa eConnect, was set up in April 2017 in the university hospital but in a different location from each ICU. Since 2017, the surgical/medical ICU (unit A) in the university hospital where the intensivists were staffed has been classified as mandatory. Other ICUs (cardiac/medical; unit B, emergency; unit C) in the university hospital and the surgical/medical ICU (unit D) in the regional hospital were classified as elective. For all patients, medical care was discussed and provided by a multidisciplinary team in each hospital. The Tele-ICU system was implemented in April 2018 after the telemedicine staff received the initial education and training of the system. The Tele-ICU physicians worked at the regional hospital ICU 1 day a week for 3 months before the implementation to create better communication.

### Program description

**Fig. 1:**
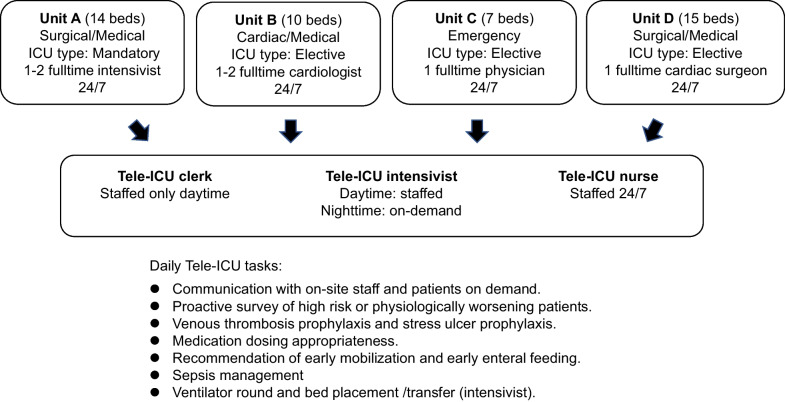
Details on on-site ICU physician coverage, the Tele-ICU staffing, and daily tasks of the Tele-ICU of Showa University Hospital

**Fig. 2:**
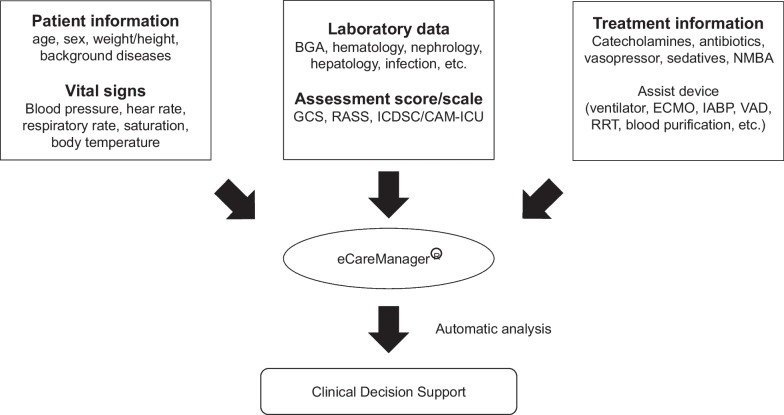
Outlines of the Tele-ICU system used in the study. *BGA* blood gas analysis, *GCS* Glasgow Coma Scale, *RASS* Richmond agitation–sedation scale, *ICDSC* Intensive Care Delirium Screening Checklist, *CAM–ICU* Confusion Assessment Method for the ICU, *NMBA* neuromuscular blocking agent, *ECMO* extracorporeal membrane oxygenation, *IABP* intra-aortic balloon pumping, *VAD* ventricular assist device, *RRT* renal replacement therapy

Details on on-site ICU physician coverage, the Tele-ICU staffing, and daily tasks of the Tele-ICU team are showed in Fig. 1. The Tele-ICU system (eCareManager® 4.1, Philips, U.S.A) used in the study supports the decision-making process by patient information centralization and real-time physiological severity evaluation based on automatic analysis (Fig. 2). The Tele-ICU staff consists of a board-certified intensivist, specially trained nurses, and a clerical assistant to the doctor. One nurse is responsible for up to 50 patients. A support center nurse is stationed 24/7. Daily Tele-ICU team tasks involve communication with on-site staff and patients using a secured audio–video system on demand and proactive survey of high risk or physiologically worsening patients to prevent unfavorable events. Venous thrombosis prophylaxis, stress ulcer prophylaxis, medication dosing appropriateness such as catecholamines, vasopressor, analgesics and sedatives, recommendation of early mobilization, early enteral feeding, and sepsis management were included in the tasks. Because the role of Tele-ICU is severity evaluation and advice in this study, the Tele-ICU physicians do not order instead of the on-site physician and only record the contents of the consultation. In addition, as the Tele-ICU physicians expertise in respiratory care and lung protective ventilation, they performed scheduled and/or on demand respiratory round. Tele-ICU physicians are given full authority of bed placement and transfer in university hospital ICU.

### Data collection and analysis

We proposed the following questions to evaluate the impact of the Tele-ICU.

1. Does the Tele-ICU implementation improve ICU performance?
2. Can the Tele-ICU maintain ICU performance longitudinally?
3. Does the expertise of the Tele-ICU physician affect ICU performance?
4. Does the Tele-ICU reduce the workload of on-site physicians?

For question 1, we conducted a pre/post study. For the analysis performed before the Tele-ICU operation, the patients were randomly selected. In brief, the study was powered to have an 80% probability for detecting a 4.5% improvement in mortality at a significance level of 0.05. From the calculation minimal enrollment target for pre-Tele-ICU was 539 cases. Fifty medical records from each quarter (3 months) in every unit were extracted to exclude the seasonal effects. In addition, at least 10 additional charts (per quarter) were extracted to account for excluded patient types, incomplete charts, or missing data. Chart extraction was performed by a clerk who did not participate in the study. We considered that 50 cases in each quarter (a total of 200 patients) from each ICU would be required for the analysis, and the exclusion rate was assumed to be 10% due to missing or invalid data. The corresponding data elements were manually abstracted from the paper medical records. In addition, the ICU and hospital mortality and LOS before and after the start of Tele-ICU were stratified by predicted hospital mortality rate by the acute physiology and chronic health evaluation (APACHE) IV score and compared. For the analysis after the Tele-ICU operation, all patients admitted to the four ICUs in the same seasonal year (from July 2018 to June 2019) were included and compared. For question 2 and 3, all adult patients registered in the Tele-ICU system between April 2018 and March 2020, just before the outbreak of the new coronavirus disease, were included in the analysis. For the evaluation of the performance of the ICU, we collected the following data elements from the medical record and the Tele-ICU system: the total number of patients, the number of intubations needed, the duration of mechanical ventilation (DOV), the number of ICU deaths, ICU mortality, hospital mortality and LOS. The APACHE IV score at ICU admission was calculated and compared for evaluation. The predicted values of mortality and LOS in the ICU and hospital and DOV were obtained from the APACHE IV score. To analyze the relationship between severity at ICU admission and mortality, the patients were divided into three groups depending on the predicted mortality by the APACHE IV score (low risk group; less than 10%, medium risk group; 10% to less than 50%, and high risk group; 50% and more). The data of all units were combined and compared to minimize the seasonal bias. For question 3, performance was compared only for patients who received invasive mechanical ventilation, because the Tele-ICU physicians specialize in respiratory care. We also investigated the topics of tele-consultation between Unit D staffs and the Tele-ICU physicians. For question 4, the frequency and total duration of access to the electronic medical record (EMR) in unit D were investigated, because physicians working in Unit D were mostly fixed and performed ICU tasks exclusively within the ICU. As a result, it was likely that the access to the EMR reflected the amount of ICU tasks. The access data of on-site physicians were stratified according to clinical career, as follows: young resident (1st and 2nd year), senior staff (16th year and above), and others (3rd to 15th year).

The study was conducted in accordance with the Declaration of Helsinki, and approved by the Human Research Ethics Committee of Showa University School of Medicine, and given the permission to use an opt out approach (Approval number 2417. Approval date; Dec 26, 2017. Study title; An evaluation of clinical efficacy of the Tele-ICU system).

### Statistical analysis

Continuous variables with parametric distributions were compared with the *t* test, and nonparametric distributions were compared with the Mann‒Whitney *U* test. Categorical data were compared using the chi-squared or Fisher’s exact test. Interrupted time series analysis and simple linear regression were used to test the significance of 2-year quarterly data. *p* < 0.05 was considered significant. Statistical analyses were performed using R (version 3.4.1).

## Results

We randomly selected 893 representative patients for the pre study between July 2015 and June 2016 in accordance with the start of Tele-ICU that was planned on July 1, 2017. However, the start of the Tele-ICU postponed until April 1, 2018 because of the delay of the implementation of related electronic system. All the collected data were valid and included. For the post study a total of 5438 patients were registered and included in the 2-year survey.

### Does the Tele-ICU implementation improve ICU performance?

The summaries and comparison of patient background and ICU performance between pre- and postintervention period are presented in Tables 1 and 2, respectively. Twenty-eight hundred and ninety-six patients of postintervention were included. Patient severity was slightly but significantly increased (*p* = 0.003). Unadjusted ICU and hospital mortality significantly decreased from 8.5 to 3.8% (*p* < 0.001) and 12.4 to 7.7% (*p* < 0.001), respectively. ICU LOS significantly decreased, but hospital LOS increased.

**Table 1:** Differences in patient background before and after Tele-ICU implementation

Variable	Jul. 2015–Jun. 2016 (*n* = 893)	Jul. 2018–Jun. 2019 (*n* = 2896)	*p* value
APACHE IV score at ICU admission, median [IQR]	45 [0, 198]	48 [6, 211]	0.003
Post elective surgery, *n* (%)	363 (40.6)	1515 (52.7)	< 0.001
Intubated, *n* (%)	173 (19.4)	386 (13.3)	< 0.001

*APACHE* acute physiological and chronic health evaluation, *ICU* intensive care unit

**Table 2:** Differences in ICU performance before and after Tele-ICU implementation

Variable	Jul. 2015–Jun. 2016 (*n* = 893)	Jul. 2018–Jun. 2019 (*n* = 2896)	*p* value
Actual ICU mortality, *n* (%)	76 (8.5)	109 (3.8)	< 0.001
Predicted ICU mortality (%), median [IQR]	1.50 [0.10, 95.10]	1.50 [0.10, 96.60]	0.467
Actual hospital mortality, *n* (%)	111 (12.4)	222 (7.7)	< 0.001
Predicted hospital mortality (%), median [IQR]	4.20 [0.00, 97.90]	4.20 [0.20, 97.70]	0.673
Actual ICU LOS (days), median [IQR]	1.62 [0.39, 66.54]	1.02 [0.00, 418.06]	< 0.001
Predicted ICU LOS (days), median [IQR]	3.41 [0.23, 12.98]	3.32 [0.11, 14.19]	0.220
Actual hospital LOS (days), median [IQR]	15.02 [0.50, 743.56]	17.01 [0.06, 418.06]	< 0.001
Predicted hospital LOS (days), median [IQR]	11.82 [1.06, 140.51]	11.83 [0.05, 64.74]	0.934

The predicted value was obtained from the APACHE IV score

*APACHE* acute physiological and chronic health evaluation, *ICU* intensive care unit, *IQR* interquartile range, *LOS* length of stay, *DOV* duration of ventilation

Table 3 shows the ICU and hospital mortality and LOS before and after the start of Tele-ICU and comparison by stratification of predicted hospital mortality rate. In the low risk group, no improvement of actual mortality was observed, whereas actual ICU LOS was significantly shortened. Contrary, actual ICU and hospital mortality rate in the medium and high risk group were significantly improved. In the high risk group, actual ICU and hospital LOS were significantly longer after the implementation. The number of high risk patients with hospital LOS of less than 5 days halved, and those with hospital LOS of 30 days or longer doubled (see Additional file 1). In addition, the mortality rate of patients whose hospital LOS was less than 5 days in high risk group remained very high; 83% before the implementation and 94% after the implementation.

**Table 3:** Differences in patient background and ICU performance before and after Tele-ICU implementation stratified by risk classification

	Low risk group			Medium risk group			High risk group		
	Jul.2015–Jun.2016	Jul.2018–Jun.2019	*p* value	Jul.2015–Jun.2016	Jul.2018–Jun.2019	*p* value	Jul.2015–Jun.2016	Jul.2018–Jun.2019	*p* value
Patient number	621	1874		186	553		86	154	
APACHE IV score at ICU admission, median [IQR]	37 [0, 93]	40 [6, 98]	< 0.001	73 [18, 128]	76 [24, 127]	0.044	119 [39, 198]	118.5 [51, 211]	0.934
Post-elective surgery, *n* (%)	320 (51.5)	1189 (63.4)	< 0.001	26 (14.0)	160 (28.9)	< 0.001	4 (4.7)	14 (9.1)	0.307
Intubated, *n* (%)	73 (11.8)	113 (6.0)	< 0.001	50 (26.9)	143 (25.9)	0.773	50 (58.1)	83 (53.9)	0.589
Actual ICU mortality, *n* (%)	3 (0.5)	4 (0.2)	0.376	22 (11.8)	32 (5.8)	0.009*	51 (59.3)	43 (27.9)	< 0.001
Predicted ICU mortality (%), median [IQR]	0.90 [0.10, 8.60]	0.90 [0.10, 8.30]	0.671	9.70 [2.40, 43.30]	8.80 [1.40, 47.80]	0.059	57.90 [27.70, 95.10]	52.35 [15.90, 96.60]	0.014
Actual hospital mortality, *n* (%)	10 (1.6)	32 (1.7)	1	38 (20.4)	71 (12.8)	0.016	63 (73.3)	68 (44.2)	< 0.001
Predicted hospital mortality (%), median [IQR]	2.30 [0.00, 9.90]	2.60 [0.20, 9.90]	0.014	20.30 [10.00, 49.90]	19.00 [10.00, 49.80]	0.173	70.80 [51.40, 97.90]	68.85 [50.10, 97.70]	0.037
Actual ICU LOS (days), median [IQR]	0.97 [0.39, 40.65]	0.91 [0.17, 418.06]	< 0.001	4.07 [0.52, 66.54]	2.91 [0.24, 67.96]	0.005	3.71 [0.41, 41.93]	6.39 [0.27, 64.04]	0.002
Predicted ICU LOS (days), median [IQR]	2.73 [0.23, 9.06]	2.71 [0.11, 8.53]	0.244	5.54 [2.00, 11.40]	5.29 [1.48, 11.15]	0.101	7.73 [4.48, 12.98]	8.11 [4.91, 14.19]	0.479
Actual hospital LOS (days), median [IQR]	14.23 [0.63, 233.04]	14.02 [0.31, 418.06]	0.429	24.91 [0.66, 202.13]	24.08 [0.30, 263.13]	0.889	5.76 [0.50, 743.56]	26.17 [0.27, 222.93]	< 0.001
Predicted hospital LOS (days), median [IQR]	11.52 [1.06, 60.14]	11.27 [0.05, 34.34]	0.753	12.06 [4.44, 50.55]	12.65 [4.54, 64.74]	0.886	12.16 [7.58, 140.51]	13.83 [7.37, 56.23]	0.277

The predicted value was obtained from the APACHE IV score. Patients were stratified by predicted hospital mortality using APACHE IV score. Predicted hospital morality of low, medium, and high risk group is less than 10%, 10–50%, and more than 50%, respectively

*APACHE* acute physiological and chronic health evaluation, *ICU* intensive care unit, *IQR* interquartile range, *LOS* length of stay, *DOV* duration of ventilation

### Can the Tele-ICU maintain ICU performance longitudinally?

**Fig. 3:**
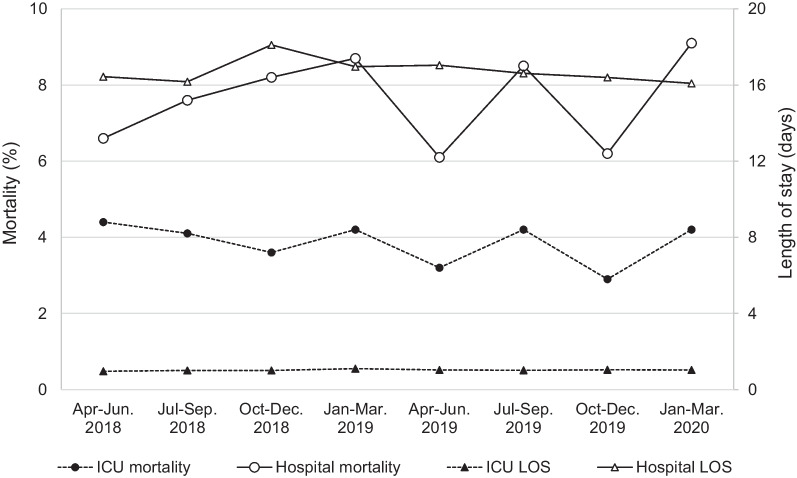
Changes in actual ICU and hospital mortality and length of stay after Tele-ICU implementation. Interrupted time series analysis was used for evaluating 2-year quarterly data

**Fig. 4:**
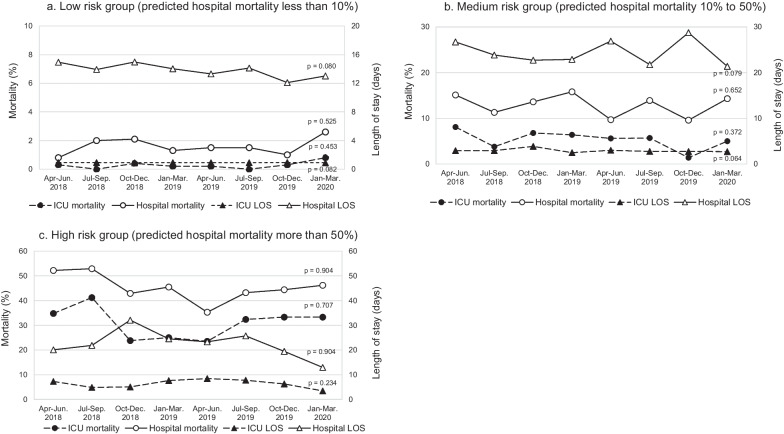
Changes in actual and predicted mortality and length of stay in **a** low, **b** median, and **c** high risk group stratified by predicted hospital mortality. Interrupted time series analysis was used for evaluating 2-year quarterly data

Quarterly data of 2 years after the implementation of the Tele-ICU are shown in Fig. 3 and Table 4. The patients were older (*p* = 0.024) and their physiological severity were significantly greater (*p* = 0.003) over time. The number of patients who required invasive mechanical ventilation increased significantly (*p* = 0.015). These results were reflected in the predicted ICU mortality, which showed significant increase, but the actual mortality was maintained around 4% over 2 years. Actual ICU and hospital mortality did not change significantly in all severity groups (Fig. 4a–c).

**Table 4:** Interrupted time series analysis of patient background and actual and predicted ICU performance after Tele-ICU implementation

Variables	Apr–Jun. 2018	Jul–Sep. 2018	Oct–Dec. 2018	Jan–Mar. 2019	Apr–Jun. 2019	Jul–Sep. 2019	Oct–Dec. 2019	Jan–Mar. 2020	*p* value
Admitted patient, *n*	547	659	753	760	724	742	584	669	0.724
Age (years), median [IQR]	70 [17, 90]	70 [16, 90]	70 [16, 90]	71 [17, 90]	70.5 [18, 90]	70 [16, 90]	72 [16, 90]	72 [17, 90]	0.024
Male, *n* (%)	306 (55.9)	409 (62.1)	467 (62.0)	459 (60.4)	465 (64.2)	461 (62.1)	364 (62.3)	418 (62.5)	0.511
APACHE IV score at admission, median [IQR]	45 [9, 177]	46 [6, 164]	49 [7, 194]	49 [7, 211]	48 [8, 159]	49 [9, 188]	50 [9, 163]	51 [9, 207]	0.003
Post elective surgery, *n* (%)	308 (58.6)	342 (52.5)	403 (53.9)	351 (46.3)	379 (52.6)	374 (51.1)	225 (40.6)	289 (43.7)	0.336
Intubated, *n* (%)	60 (11.0)	66 (10.0)	115 (15.3)	89 (11.7)	116 (16.0)	127 (17.1)	100 (17.1)	139 (20.8)	0.015
ICU performance									
Actual ICU mortality, *n* (%)	24 (4.4)	27 (4.1)	27 (3.6)	32 (4.2)	23 (3.2)	31 (4.2)	17 (2.9)	28 (4.2)	0.785
Predicted ICU mortality (%), median [IQR]	1.10 [0.10, 96.20]	1.20 [0.10, 93.40]	1.65 [0.10, 96.60]	1.85 [0.10, 96.20]	1.50 [0.10, 91.10]	1.60 [0.10, 93.80]	1.90 [0.10, 90.90]	1.80 [0.10, 97.30]	0.022
Actual hospital mortality, *n* (%)	36 (6.6)	50 (7.6)	62 (8.2)	66 (8.7)	44 (6.1)	63 (8.5)	36 (6.2)	61 (9.1)	0.631
Predicted hospital mortality (%), median [IQR]	3.35 [0.20, 98.50]	3.50 [0.30, 95.60]	4.25 [0.20, 97.10]	4.90 [0.20, 97.70]	4.20 [0.20, 95.10]	3.85 [0.20, 98.80]	5.00 [0.30, 86.20]	4.65 [0.20, 97.70]	0.056
Actual ICU LOS (days), median [IQR]	0.96 [0.00, 77.86]	1.00 [0.00, 48.61]	1.00 [0.01, 67.96]	1.13 [0.00, 418.06]	1.03 [0.04, 48.24]	1.01 [0.00, 48.54]	1.04 [0.03, 33.76]	1.03 [0.01, 78.77]	0.869
Predicted ICU LOS (days), median [IQR]	3.08 [0.04, 14.57]	3.10 [0.17, 11.13]	3.37 [0.17, 12.02]	3.41 [0.17, 14.19]	3.34 [0.11, 11.54]	3.32 [0.17, 13.23]	3.32 [0.14, 12.27]	3.39 [0.17, 11.77]	0.057
Actual hospital LOS (days), median [IQR]	16.44 [0.09, 401.70]	16.17 [0.06, 417.74]	18.10 [0.09, 418.06]	16.96 [0.27, 418.06]	17.04 [0.06, 417.74]	16.61 [0.09, 333.09]	16.39 [0.03, 238.80]	16.08 [0.12, 333.09]	0.193
Predicted hospital LOS (days), median [IQR]	12.61 [0.65, 60.97]	12.23 [1.38, 47.14]	12.47 [1.72, 55.78]	11.17 [0.05, 56.23]	11.29 [0.17, 64.74]	11.66 [0.11, 57.21]	10.53 [1.48, 54.06]	10.77 [0.43, 57.94]	0.004

The predicted value was obtained from the APACHE IV score. Interrupted time series analysis was used for evaluating 2-year quarterly data

*APACHE* acute physiological and chronic health evaluation, *ICU* intensive care unit, *IQR* interquartile range, *LOS* length of stay, *DOV* duration of ventilation

### Does the expertise of the Tele-ICU physician affect ICU performance?

We investigated the ICU performance in mechanically ventilated patients in 2 years after the implementation of the Tele-ICU (Table 5). The number and proportion of intubated patients increased from 330 (52.9%) in 2018 to 482 (70.0%) in 2019. Actual ICU mortality unchanged, while actual hospital mortality significantly reduced. Actual DOV significantly decreased from 3.00 [1.00, 30.00] days to 2.00 [1.00, 30.00] days (*p* = 0.007). Mechanical ventilation-related issues were the most frequent topic of consultation from Unit D staff to the Tele-ICU physicians. The content of consultation included respiratory management (63.6%), order confirmation (7.6%), order alteration/modification (6.8%), and patient care-related (22.0%). Details in respiratory management consultation included ventilation settings (44.7%), ventilator/ECMO weaning (21.1%), sedatives (15.8%), early rehabilitation (1.3%), and others (17.1%).

**Table 5:** Differences in patient background and ICU performance of mechanically ventilated patients in the 1st and 2nd years after Tele-ICU implementation

Variable	1st year	2nd year	*p* value
Patient number	624	689	
Age (years), median [IQR]	72.5 [20, 90]	72.0 [16, 90]	0.314
Male, *n* (%)	375 (60.1)	460 (66.8)	0.014
APACHE IV score at ICU admission, median [IQR]	75 [7, 211]	79 [8, 207]	0.033
Post-elective surgery, *n* (%)	261 (41.9)	304 (44.8)	0.288
Intubated, *n* (%)	330 (52.9)	482 (70.0)	< 0.001
Actual ICU mortality, *n* (%)	73 (11.7)	65 (9.4)	0.207
Predicted ICU mortality (%), median [IQR]	11.00 [0.10, 96.60]	12.00 [0.10, 97.30]	0.309
Actual hospital mortality, *n* (%)	121 (19.4)	98 (14.2)	0.014
Predicted hospital mortality (%), median [IQR]	15.90 [0.20, 98.50]	18.80 [0.20, 98.80]	0.353
Actual ICU LOS (days), median [IQR]	4.58 [0.04, 115.85]	3.81 [0.00, 48.54]	0.277
Predicted ICU LOS (days), median [IQR]	6.47 [1.08, 14.57]	6.62 [0.72, 13.23]	0.192
Actual hospital LOS (days), median [IQR]	25.38 [0.09, 417.74]	27.18 [0.03, 333.09]	0.165
Predicted hospital LOS (days), median [IQR]	13.72 [4.69, 56.23]	14.81 [3.36, 64.74]	0.045
Actual DOV (days), median [IQR]	3.00 [1.00, 30.00]	2.00 [1.00, 30.00]	0.007
Predicted DOV (days), median [IQR]	3.43 [0.17, 10.77]	3.71 [0.30, 12.60]	0.081

The predicted value was obtained from the APACHE IV score

*APACHE* acute physiological and chronic health evaluation, *ICU* intensive care unit, *IQR* interquartile range, *LOS* length of stay, *DOV* duration of ventilation

### Does the Tele-ICU reduce the workload of on-site physicians?

**Fig. 5:**
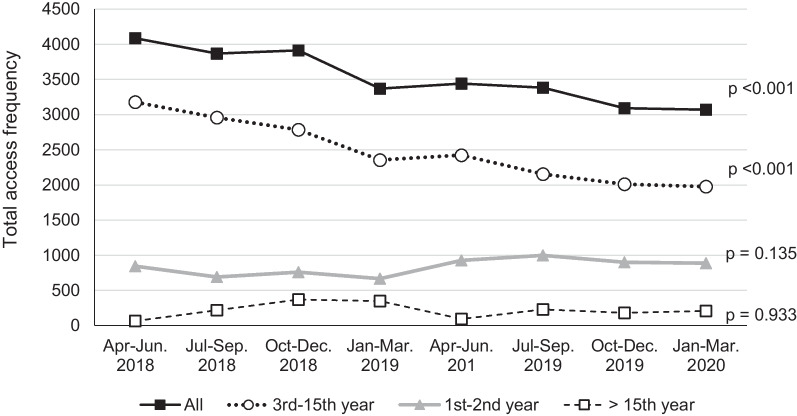
Changes in the total frequency of access to the electronic medical record by on-site physicians in unit D stratified by clinical careers. Interrupted time series analysis was used for evaluating 2-year quarterly data

**Fig. 6:**
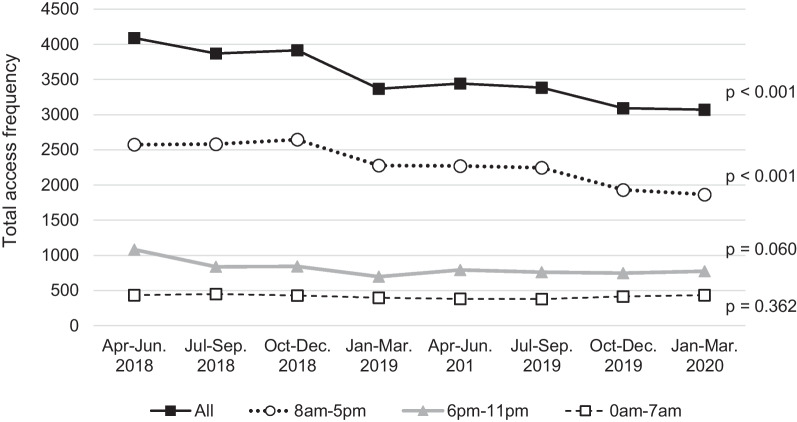
Changes in the total frequency of access to the electronic medical record by on-site physicians in unit D stratified by working hour. Interrupted time series analysis was used for evaluating 2-year quarterly data

**Fig. 7:**
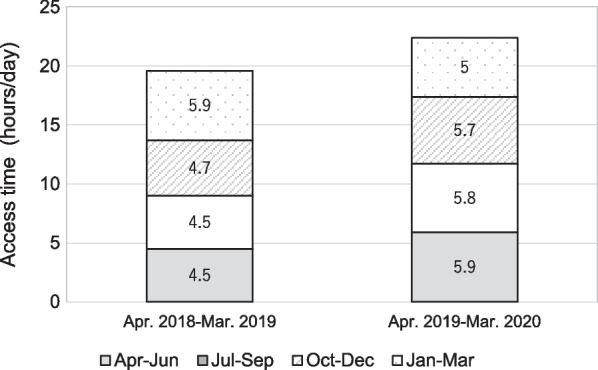
Changes in total daily access duration (median) of Tele-ICU physicians to the electrical medical record in Unit D

We investigated the total frequency and duration of access to the EMR to evaluate the workload of the on-site staff in unit D as well as the Tele-ICU physician. The log-ins of the on-site physicians continuously and significantly (*p* < 0.001) decreased during the 2-year operation and were decreased by 25% compared to the initial count (Fig. 5). Notably, the log-ins of physicians with 3–15 years of clinical experience decreased (*p* < 0.001), while those with other clinical career strata showed no change (Fig. 5). When the access frequency was stratified by working hours, only access during the daytime shift decreased significantly (Fig. 6). Total access time per day for on-site physicians remained unchanged. The number of patient admission in 2019 decreased by about 10% compared to 2018, resulted from the decrease in elective surgery patients who did not require postoperative respiratory management. Changes in the median daily access duration of the Tele-ICU physician to the EMR for Unit D patients are shown in Fig. 7. The annual access duration in 2019 was longer than that in 2018, and the access duration in each quarter in 2019 was longer than that in 2018, except for the results between January and March, but the difference did not reach statistical significance.

## Discussion

Tele-ICU system used in the study is developed in the United States. There is no similar system developed in Japan yet. To use the system, educated staff are required, and there are differences between Japan and the United States in terms of job authority and legal issues. Since these differences can become obstacles in system operation, we conducted the study whether we could obtain the results similar to previous studies in the U. S. Our study confirmed favorable impacts of the Tele-ICU on ICU and hospital mortality, with sustained effects. We also found the positive effect on the performance of the mechanically ventilated patients. Furthermore, our study showed that on-site physicians spent significantly less time on EMR-related tasks after the Tele-ICU implementation.

In our data, mortality and LOS after the Tele-ICU implementation were expected to increase as a result of a significant increase in patient severity because of a decrease in patients undergoing elective surgery and a 1.5-fold increase in the need for mechanical ventilation. However, the results showed a reduction in unadjusted ICU and hospital mortality. In addition, stratified by predicted hospital mortality, the ICU and hospital mortality significantly improved in the medium and high risk groups, where the Tele-ICU intervenes more frequently. Our data showed actual ICU mortality was affected by increased patient severity but remained stable in range of 4.2–4.4% in 2 years. The reason of increased LOS in high risk patients is likely that the treatment of saved high risk patients took longer time. We believe these outcomes resulted from the effective implementation of the Tele-ICU service in the elective ICU through providing adequate monitoring and care. In previous pre/post studies, the effects of the Tele-ICU have varied. Studies showed that the implementation of the Tele-ICU led to a significant reduction in ICU and hospital mortality and LOS and that these improvements were associated with the standardization of care [9–11]. Another study showed that there was no effect on survival or LOS, and the adjusted analysis did not change the results [12]. For some ICUs, low-intensity models such as night coverage may be sufficient, but practices that require a positive approach to others, especially care, may benefit from the high-intensity model [13]. It has been reported that the successful utilization of the Tele-ICU resources to provide adequate care to high-acuity patients is dependent on the ability of health care systems [14]. A retrospective study showed that in-hospital mortality in a mandatory ICU was significantly lower than that in a high care unit or a step-down unit [15]. In Japan, beds that are equipped and can be used for intensive care exist in the high care unit and the emergency department [5]. However, all regions are not able to use those as intensive care beds due to a lack of intensivists. Considering that one-fourth of ICUs in Japan are classified as elective, our results indicate that the Tele-ICU is effective in improving mortality in these ICUs. A successful Tele-ICU implementation will be a feasible goal to make the elective ICU more similar to the mandatory ICU without increasing the current resource.

Notably, DOV was significantly shortened in our results (Table 5). It has been reported that implementation of Tele-ICU ventilator rounds was associated with improved adherence to lung protective ventilation and significant reductions in both DOV and ICU mortality [16]. This study also suggested that the implementation of the Tele-ICU system will be used to help delegate standard care related to respiratory management to the on-site staff. In addition, all Tele-ICU physicians are board-certified intensivists and have expertise in mechanical ventilation management, while the on-site physician does not always have this expertise. The most common bedside consultations were on topics related to ventilation settings, ventilator weaning, sedation, and early rehabilitation. The optimal structure of the Tele-ICU team is one that leverages expert clinical knowledge to address the needs of critically ill patients, regardless of hospital location or availability of on-site staff [17]. From this point of view, it is suggested that the Tele-ICU system effectively utilized the expertise of the Tele-ICU physician.

Methods for measuring intensive care physician workload have not been standardized. Recently it is reported that the use of EMR is directly associated with physician fatigue and burnout [7, 8]. EMRs’ documentation and related tasks were identified as one of the main causes of physician burnout [18]. In our results, the frequency of access to the EMR decreased after the implementation of the Tele-ICU; however, the Tele-ICU physicians' access tended to increase. The result that access to the EMR decreased by 25% is noteworthy. It is difficult to explain the reason for the decrease in EMR access by the smaller yearly patient admission, because the patient number other than elective surgery patients did not change. The decrease in access was seen only in the daytime shift, which was compatible with the working hours of the Tele-ICU physician. Interestingly, access by physicians with 3–15 years of clinical experience significantly decreased, but there was no such change for physicians of other career stages. It is unclear how much reducing EMR-related task contributes to reducing the on-site physician’s workload. Because procedures and orders are immediately documented in the EMR, total access reduction suggests less intervention and stable condition of ICU patients. These results indicate that the Tele-ICU promoted work sharing between the Tele-ICU and the on-site staff, demonstrating that the Tele-ICU could be a solution of the shortage of intensivist as well as the regional disparities.

Our study has several limitations. This was an observational study and thus has an increased risk of confounders. This study was conducted in a single healthcare system, and extrapolation to other systems or settings should be done carefully. Our system is a continuous care model, and it is unclear how other models, such as scheduled or responsive care models, would be affected. The mortality rate was a key outcome in the study, but the final decision on treatment was made by the on-site physician. We used unadjusted data for the assessment.

## Conclusions

Our study showed the implementation of the Tele-ICU was associated with lower mortality, especially in medium and high risk patients, and decreased EMR-related tasks of on-site physicians. These results suggests that the Tele-ICU could be one of the solutions of the shortage of intensivists and regional disparities for intensive care.

## Supplementary Information

**Additional file 1.** Distribution of LOS in high risk patients before and after the Tele-ICU implementation. LOS; length of stay (days).

## Data Availability

The data sets generated during and/or analyzed in the current study are not publicly available due to patient confidentiality.

## Abbreviations

Tele-ICU: Telemedicine intensive care unit
ICU: Intensive care unit
LOS: Length of stay
DOV: Duration of mechanical ventilation
APACHE: Acute physiology and chronic health evaluation
EMR: Electronic medical record
BGA: Blood gas analysis
GCS: Glasgow Coma Scale
RASS: Richmond agitation–sedation scale
ICDSC: Intensive Care Delirium Screening Checklist
CAM–ICU: Confusion Assessment Method for the ICU
NMBA: Neuromuscular blocking agent
ECMO: Extracorporeal membrane oxygenation
IABP: Intra-aortic balloon pumping
VAD: Ventricular assist device
RRT: Renal replacement therapy
IQR: Interquartile range

## References

[1] Yoo BK, Kim M, Sasaki T, Melnikow J, Marcin JP (2016). Economic evaluation of telemedicine for patients in ICUs. Crit Care Med.

[2] Fusaro MV, Becker C, Scurlock C (2019). Evaluating Tele-ICU implementation based on observed and predicted ICU mortality: a systematic review and meta-analysis. Crit Care Med.

[3] Chen J, Sun D, Yang W, Liu M, Zhang S, Peng J, Ren C (2018). Clinical and economic outcomes of telemedicine programs in the intensive care unit: a systematic review and meta-analysis. J Intensive Care Med.

[4] Lilly CM, Cody S, Zhao H, Landry K, Baker SP, McIlwaine J, Chandler MW, Irwin RS, University of Massachusetts Memorial Critical Care Operations G (2011). Hospital mortality, length of stay, and preventable complications among critically ill patients before and after Tele-ICU reengineering of critical care processes. JAMA.

[5] Survey of the number of beds in ICUs and treatment units equivalent to ICU by prefecture. https://www.jsicm.org/news/upload/icu_hcu_beds.pdf.

[6] ICU Functional Assessment Committee (2021). A survey report of intensivist training certified facilities, 2020. J Jpn Soc Intensive Care Med.

[7] Khairat S, Coleman C, Ottmar P, Jayachander DI, Bice T, Carson SS (2020). Association of electronic health record use with physician fatigue and efficiency. JAMA Netw Open.

[8] Downing NL, Bates DW, Longhurst CA (2018). Physician burnout in the electronic health record era: are we ignoring the real cause?. Ann Intern Med.

[9] Becker CD, Fusaro MV, Scurlock C (2019). Telemedicine in the ICU: clinical outcomes, economic aspects, and trainee education. Curr Opin Anaesthesiol.

[10] Becker CD, Fusaro MV, Al Aseri Z, Millerman K, Scurlock C (2020). Effects of telemedicine ICU intervention on care standardization and patient outcomes: an observational study. Crit Care Explor.

[11] Fusaro MV, Becker C, Miller D, Hassan IF, Scurlock C (2021). ICU telemedicine implementation and risk-adjusted mortality differences between daytime and nighttime coverage. Chest.

[12] Nassar BS, Vaughan-Sarrazin MS, Jiang L, Reisinger HS, Bonello R, Cram P (2014). Impact of an intensive care unit telemedicine program on patient outcomes in an integrated health care system. JAMA Intern Med.

[13] Caples SM (2019). Intensive care unit telemedicine care models. Crit Care Clin.

[14] Lilly CM, Mickelson JT (2019). Evolution of the intensive care unit telemedicine value proposition. Crit Care Clin.

[15] Ohbe H, Sasabuchi Y, Yamana H, Matsui H, Yasunaga H (2021). Intensive care unit versus high-dependency care unit for mechanically ventilated patients with pneumonia: a nationwide comparative effectiveness study. Lancet Reg Health West Pac.

[16] Kalb T, Raikhelkar J, Meyer S, Ntimba F, Thuli J, Gorman MJ, Kopec I, Scurlock C (2014). A multicenter population-based effectiveness study of teleintensive care unit-directed ventilator rounds demonstrating improved adherence to a protective lung strategy, decreased ventilator duration, and decreased intensive care unit mortality. J Crit Care.

[17] Welsh C, Rincon T, Berman I, Bobich T, Brindise T, Davis T (2019). TeleICU Interdisciplinary Care Teams. Crit Care Clin.

[18] Muhiyaddin R, Elfadl A, Mohamed E, Shah Z, Alam T, Abd-Alrazaq A, Househ M (2022). Electronic health records and physician burnout: a scoping review. Stud Health Technol Inform.

