# Supplementary material for: An evaluation of the impact of the implementation of the Tele-ICU: a retrospective observational study
Source: J Intensive Care. 2023 Mar 7;11:9. doi: 10.1186/s40560-023-00657-4 (PMC9989570; doi:10.1186/s40560-023-00657-4)
Supplement: Supplementary file 1 — Additional file 1. Distribution of LOS in high risk patients before and after the Tele-ICU implementation. LOS; length of stay (days). [file 40560_2023_657_MOESM1_ESM.pptx]

## Slide 1
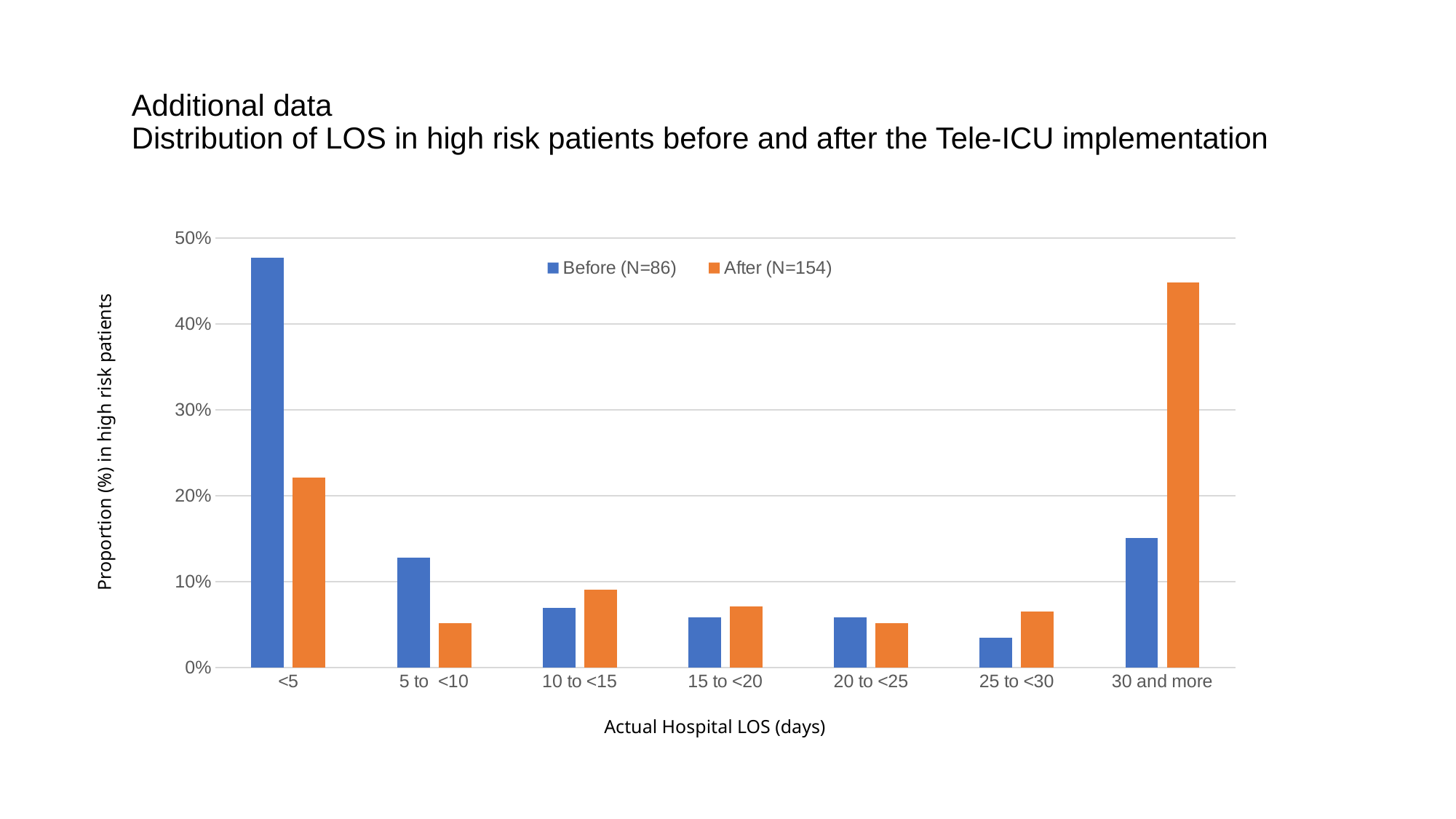

# Additional data Distribution of LOS in high risk patients before and after the Tele-ICU implementation
### Chart
| Category | Before (N=86) | After (N=154) |
|---|---|---|
| <5 | 0.47674418604651164 | 0.22077922077922077 |
| 5 to <10 | 0.12790697674418605 | 0.05194805194805195 |
| 10 to <15 | 0.06976744186046512 | 0.09090909090909091 |
| 15 to <20 | 0.05813953488372093 | 0.07142857142857142 |
| 20 to <25 | 0.05813953488372093 | 0.05194805194805195 |
| 25 to <30 | 0.03488372093023256 | 0.06493506493506493 |
| 30 and more | 0.1511627906976744 | 0.44805194805194815 |Proportion (%) in high risk patients
Actual Hospital LOS (days)
